# Ovarian Collision Tumor, Massive Mucinous Cystadenoma, and Benign Mature Cystic Teratoma

**DOI:** 10.7759/cureus.16221

**Published:** 2021-07-06

**Authors:** Abdullah M Alayed, Abdullah S Almawi, Ebtehaj G Alghamdi, Hana S Alfaleh, Nouf S Kadasah

**Affiliations:** 1 Radiology, Prince Sultan Military Medical City, Riyadh, SAU; 2 College of Medicine, Bisha University, Bisha, SAU; 3 College of Medicine, Princess Nourah Bint Abdulrahman University, Riyadh, SAU; 4 Radiology, King Abdulaziz Medical City, Riyadh, SAU

**Keywords:** ovarian collision tumor, massive mucinous cystadenoma, benign mature cystic teratoma, collision tumors, ovarian tumors

## Abstract

Collision tumors are rare neoplasms defined by the presence of two distant tumors in the same organ without any histological intermixing. Ovarian tumors are often asymptomatic during the early stages and become symptomatic when increased in size causing vague abdominal pain, abdominal distention, vomiting, and frequent urination. We report here a case of a 28-year-old female who presented with a history of worsening abdominal pain and distension. An abdominopelvic ultrasound scan showed a huge complex mass occupying the pelvic area with non-visualization of the left ovary suggesting an ovarian origin; further characterization by cross-sectional imaging by both CT and MRI were performed confirming a left ovarian complex mass containing multiseptated cystic and fat component at the same time along with massive ascites. After surgical resection of the mass, histopathology revealed mucinous cystadenoma coexisting with cystic teratoma.

## Introduction

A collision tumor is defined as the coexistence of two different tumors within the same organ with no histological intermixing. Collision tumors have been described in various organs including the stomach, esophagus, liver, kidney, bone, lungs, and brain but once it comes to the ovaries; they are rare [1]. Teratoma is one of the most common collision combinations in the ovaries. Ovarian teratomas are the most common ovarian tumors especially benign mature cystic teratomas which are known as dermoid cysts and represent 12%-15% [2].

Ovarian mucinous cystadenoma represents approximately 80% of the ovarian mucinous neoplasm and 20%-25% of all types of benign ovarian tumors. Generally, mucinous cystadenomas are larger than serous cystadenomas and they are rare to present bilaterally (2%-5%) [3]. In this case report, we present a case of a 28-year-old female who presented with a history of worsening abdominal pain and distension which finally was diagnosed as a left ovarian complex mass containing multiseptated cystic and fat components at the same time along with massive ascites that histopathology revealed mucinous cystadenoma coexisting with cystic teratoma.

## Case presentation

A 28-year-old, nulligravid lady, presented with abdominal pain and abdominal distension. On examination, her vital signs were normal and she was clinically stable with normal general physical examination apart from a palpable pelvic mass. She was initially investigated with an abdominopelvic ultrasound scan and serum human chorionic gonadotropin (hCG) test to exclude pregnancy.

**Figure 1:**
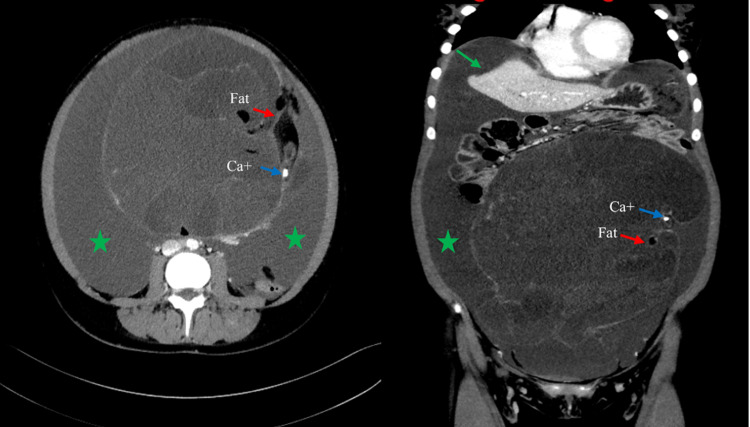
Axial and coronal CECT of the abdomen and pelvis showing a large multilocular cystic left adnexal mass with multiple fat containing lesion (red arrow) and foci of calcification (blue arrow) on the left aspect of the mass. Associated with massive ascites (green star) that is causing scalloping on the liver surface (green arrow). CECT, contrast-enhanced computed tomography

Her ultrasound scan revealed a massive 21 cm x 11 cm abdominopelvic mass originating from the left ovary, it was a complex multiseptated cystic mass associated with massive ascites; her carbohydrate antigen (CA) 125 level was 94 U/mL (normal, 0-35 U/mL), CA19.9 was 182 U/mL (normal, 0-34 U/mL, according to our hospital), human chorionic gonadotropin (hCG) was 0 IU/L, AFP was 1.2 KIU/L (normal, 0-5.8 KIU/L), and CEA was 83.9 (normal, 0-3.4 ug/L). Contrast‐enhanced CT showed a large midline cystic multilocular left adnexal mass with few foci of macroscopic fat component and calcification, the septa were thick. It was measuring 28 cm x 16.8 cm x 20 cm [transverse, longitudinal, and craniocaudal (CC) view respectively]. Also, there were associated large ascites causing scalloping on the liver surface (Figure 1).

**Figure 2:**
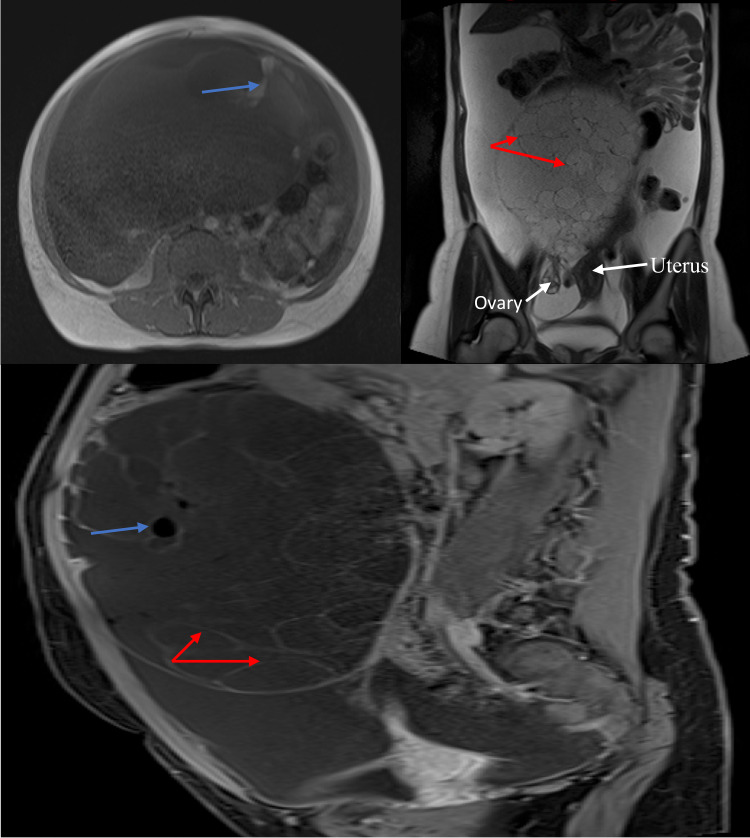
Axial, coronal, and sagittal MRI of the abdomen and pelvis showing large left ovarian multilocular cystic mass, see the enhancing septations (red arrows). The macroscopic fat shows bright signal in the non-fat saturated T2 FSE with corresponding drop in signal in the T1 fat suppressed post-contrast image (blue arrow). FSE, fast spin echo

Abdominal MRI revealed the same findings of the huge left ovarian mass with thick enhanced septa, containing fat and few foci of calcification. The cystic part of the mass showed dark T1, bright T2 signal intensity, the fatty component was bright on the non-fat saturated T1 and T2 weighted images and suppressed on T1 fat-saturated pre-contrast images. The right ovary was normal. Along with the massive ascites scalloping the liver, there were multiple- peritoneal deposits, the largest measured 1.8 cm (Figure 2).

The patient went for open surgery for left adnexectomy, omentectomy, and appendectomy. On histopathology, the ovarian mass was confirmed to be a mucinous cystadenoma with focal epithelial proliferation mixed with mature cystic teratoma, negative for malignancy. The omentum showed fibroconnective tissue with acellular mucin, negative for malignancy. And the appendix was normal without dysplasia or malignancy. The peritoneal deposits were sent for histopathology as well which showed multiple foci of acellular mucinous deposition with exuberant inflammation. And the diagnosis of collision tumor was made based on the radiological and histopathological findings.

## Discussion

Collision tumors are uncommon neoplasms that represent two distant tumors in the same organ without any histological intermixing. Collision tumors have been reported in different organs such as the gastrointestinal tract, central nervous system, lung, skin, adrenals, etc. However, collision tumors are uncommon to involve the ovary. Collision tumor is more common to be unilateral, and its size ranges from 5.5 to 200 cm, and mostly the patients' ages range from 17 to 66 years [4]. Teratoma is considered one of the most common components of collision combination in the ovary.

Cystic teratoma is the most common ovarian germ cell tumor representing 20% of all the ovarian tumors in adults, and it is the most common benign ovarian tumor among women less than 45 years old [5]. On gross appearance, mature cystic teratoma is characterized by a unilocular tumor filled with sebaceous material [6]. CT and MRI are the most sensitive modalities to diagnose mature cystic teratomas [7]. On CT, the diagnostic findings of mature cystic teratoma are seen as fat attenuation within a cyst, with or without calcification in the wall. MRI findings of mature cystic teratomas are seeing sebaceous content of dermoid as high signal intensity on T1-weighted images [8].

Ovarian epithelial neoplasms represent around 60% of all ovarian tumors and 40% of benign ovarian tumors. Ovarian cystadenomas are common benign epithelial neoplasms and represent less than 5% of all epithelial ovarian malignancies [9]. They are subdivided histologically into serous and mucinous epithelial neoplasm. Ovarian cystadenomas are commonly encountered in women aged between third and fifth decades, and rarely seen among adolescents [10].

Ovarian mucinous cystadenomas usually manifest as unilateral, multilocular, cystic, and ovarian lesions [11]. The most common form of ovarian collision tumors in the literature is the combination of teratoma and mucinous cystadenoma similar to our case but something unusual about our scenario is the late presentation with massive mucinous ascites along with nonmalignant peritoneal deposits [12].

## Conclusions

In conclusion, the most common form of ovarian collision tumors in the literature is the combination of teratoma. Although ovarian collision tumors are extremely rare, the coexistence with other benign ovarian tumors is rarer. Multiloculated cysts have to be extensively examined radiologically and histopathologically, not to miss any component which might have a bearing on prognosis of the patient.
